# Intra-operative dosimetry of trans-rectal ultrasound guided ^125^I prostate implants using C-arm fluoroscopic images

**DOI:** 10.4103/0971-6203.26689

**Published:** 2006

**Authors:** Paul B. Ravindran, C. Lewis, J. Van Dyk, D. D'Souza

**Affiliations:** Department of Radiation Oncology, Christian Medical College & Hospital, Vellore, Tamil Nadu, India; * Department of London Regional Cancer Program, London Health Sciences Centre and the University of Western Ontario, London, Ontario, Canada

**Keywords:** ^125^I, C-arm fluoroscopy, prostate implant, trans-rectal ultrasound

## Abstract

Permanent implantation of radioactive seeds is a viable and effective therapeutic option widely used today for early-stage prostate cancer. The implant technique has improved considerably during the recent years due to the use of image guidance; however, real-time dose distributions would allow potential cold spots to be assessed and additional seeds added. In this study, we investigate the use of a conventional C-arm fluoroscopy unit for image acquisition and evaluation of dose distribution immediately after the implant. The phantom study indicates that it is possible to obtain seed positions within ±2 mm. A pilot study carried out with three patients indicated that it is possible to obtain seed positions and calculate the dose distribution with C-arm fluoroscopy and about 95% of the seeds were reconstructed within ±2 mm. The results could be further improved with better digital imaging.

Brachytherapy with permanent implant of ^125^I seeds is considered standard therapy and is an alternative to radical prostatectomy or EBRT for patients with localized prostate adenocarcinoma. This is used as monotherapy or in combination with external beam radiotherapy (EBRT). With the use of trans-rectal ultrasound (TRUS) guidance, it has become possible to accurately implant radioactive seeds into the prostate with image guidance. Commonly accepted indications for prostate permanent implant as monotherapy include localized prostate cancer of clinical Stage Tlc or T2a, prostate specific antigen (PSA) less than or equal to 10, Gleason Score less than or equal to 6, prostate volume less than 50 cc, no nodal or distant metastases and no prior trans urethral resection of prostate (TURP).[1,2] Our procedure for permanent implant of the prostate is performed in four steps: a TRUS volume study, pre-planning to decide on the geometry of implant and the number of seeds, implantation of seeds on the day of implant and a CT- or MR-based post plan at about 4 weeks after implant. The images obtained during the TRUS volume study performed a few weeks prior to implant are used for planning. The plan results in a 3D conformal dose distribution that delivers a high dose of radiation to the gland with relative sparing of the surrounding normal structures (rectum and bladder). Under TRUS guidance with a perineal template, about 100 seeds are deposited based on the treatment plan. However, the geometry of the implant achieved is never the same as the geometry of the implant planned. Sources may not be placed in the intended location due to many factors including needle deviation, gland deformation, source migration and gland swelling. CT-based post-planning is performed around day 30 post-implant and at this point, an undesired cold spot may be discovered. The value of “fixing” this is uncertain and requires additional resources. Options include another brachytherapy procedure to deposit additional seeds or supplemental external beam radiation.

Real-time dosimetry would be ideal to allow for a rapid evaluation of the implant quality intra--operatively and allow the correction of cold spots. Such a system would provide the dose distribution as the seeds are deposited. However, this would require an imaging system that is both real-time and provides clear information on the three-dimensional position of the seeds deposited. Attempts have been made[3] to use 3D ultrasound images to obtain real-time dosimetry, but obtaining the exact seed coordinates from ultrasound images is difficult due to the poor contrast of seeds. Use of interventional MRI has also been reported for real-time dosimetry of prostate implants,[4] but this technology is not commonly available in brachytherapy procedure rooms. A few authors have used radiotherapy simulators[5,6] that are isocentrically mounted to obtain the seed positions accurately with radiographic images taken at AP and two oblique angles. In this study, we have investigated the use of a C-arm fluoroscopy unit for intra-operative dosimetry of prostate implants.

## Materials and Methods

The prostate implants were performed with a Siemens template under TRUS guidance. ^125^I seeds (model 6711) of Amersham Medi-Physics Inc. were used for most of the implants. Here we explore the use of a ceiling fluoroscopy unit with a C-arm for intra-operative dosimetry of prostate implants. To obtain the seed positions accurately for 3D reconstruction of the implant from radiographic images, the best-known method has been the three-film method.[5–8] This method requires radiographs of the implant at two oblique angles and at an AP projection. The minimum oblique angles reported in the literature are +8° and −8°. However, to minimize the reconstruction error, an angle of 45° is recommended. In this study, we have investigated the use of C-arm for intra-operative dosimetry of the seed implant with the three-film technique. In order to standardize the method and to validate the software developed for this purpose, initially phantom studies were performed with a Perspex phantom and a Gel phantom. The software was also evaluated on the images obtained for three patients for reconstructing the seed geometry and for obtaining dose distributions.

### Image acquisition

**Figure 1:**
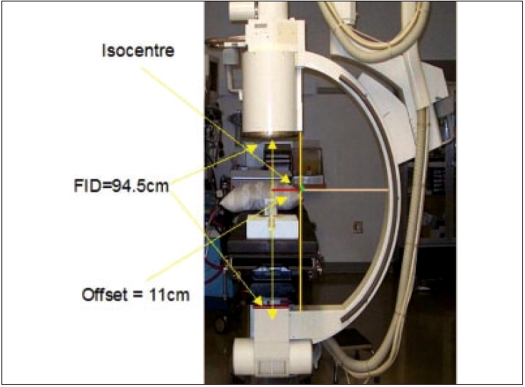
C-arm X-ray unit used in the study, the offset between the position of the centre of rotation and the X-ray beam central axis is illustrated. (FID - focus to -image intensifier distance)

The images were directly acquired from the Siemens fluoroscopy unit using a high-resolution DT 3162 video frame grabber from *Data Translation.* A personal computer (PC) with the DT 3162 video frame grabber was connected to the Siemens fluoroscopy unit and the images were acquired directly to the PC through a composite video input of the frame grabber. Three images, viz, Right Posterior Oblique (RPO), posterior-anterior (PA) and Left Posterior Oblique (LPO) were acquired at −8°, 0° and +8°. Using the C-arm, these were the maximum angles that could be achieved due to the limitation in the rotation of the C-arm along the transverse axis of the patient. Since the central axis of the X-ray source does not pass through the center of rotation of the C-arm, a coordinate transformation was required. The C-arm fluoroscopy unit is shown in Figure 1, with its rotational geometry marked.

The software developed has modules for automatic extraction of seed position, reconstruction of the implant geometry, determination of dose as per the American Association of Physicists in Medicine (AAPM) TG-43 protocol[9] and for display of dose distribution.

### Automatic extraction of seed position

The first step in obtaining the 3D reconstructed source position is to input the seed coordinates from all three radiographs. Since manual digitization would be time consuming and difficult, a software module was developed for automatic extraction of the seed positions from the radiographs. The method adopted here is as suggested by Tubic *et al*.[7] Automatic extraction of seed coordinates from the radiographs is a difficult task as the background image such as the bone could introduce detection of false seeds. Hence the initial task is to remove the background and convert the image to binary image containing only the seeds. This is performed by fast removing the background using morphological operation called ‘top-hat opening.’ Top-hat opening provides the image minus the morphological opening of the image and thus removes the background. A binary image containing the seeds is obtained from this image by automatic gray level threshold selection. A ‘connected component labeling’ method is used to obtain the seed coordinates from this binary image. These steps are repeated for all the three images. The software may not identify the seeds that are overlapping as two different seeds and hence provision is given for manual intervention to correct for false detection.

### Seed reconstruction and dose distribution

#### Reconstruction of the implant geometry:

The geometry of the implant is reconstructed from the extracted seed positions in 3D space. Since the seeds extracted in each film will not be in the same order, the task will be to match the corresponding seed positions obtained from all the three films. The three-film technique has been used for several years for reconstruction of the implant geometry from the extracted seed coordinates. More recently, relatively accurate reconstruction methods such as Fast Cross projection reconstruction;[10] operator-free, film-based reconstruction;[11] reconstruction using Hough trajectories;[12] seed reconstruction from incomplete data set[13]; and seed reconstruction using simulated annealing[7] have been reported. In this study, we have used the method suggested by Tubic *et al*[7] for the 3D reconstruction of the implant geometry.

A program was developed in Visual C for reconstruction of prostate implants using simulated annealing suggested by Tubic *et al*.[8] Simulated annealing is a powerful optimization technique that can determine the global minimum for problems where multiple local minimum values exist. It keeps a variable temperature (T) that determines the behavior of the annealing process. This variable T will be initialized to a very large value at the beginning and will be gradually decreased (cooling down) till a minimum value is obtained. For details on the use of simulated annealing for matching the seeds, the readers may refer to Tubic *et al*.[8]

Since the seed positions were extracted from a conventional C-arm fluoroscopy unit, which is non-isocentric, coordinate transformations are needed before reconstructing the implant geometry. The geometry of the C-arm unit and the offset of the beam central axis from the center of rotation of the C-arm are illustrated in Figure 1. A dose engine that calculates the dose as per the TG43 formalism assuming point source model was also incorporated to display the dose distribution.

### Phantom studies

In order to validate the use of C-arm and the software developed for real-time dosimetry of seed implant, an in-house perspex phantom was developed. The phantom was made of acrylic of size 8 cm^3^ with seed placement holes provided to the geometry of the template used for implant. About 104 dummy seeds were placed in the phantom in a triangular geometry. RPO, PA and LPO images as mentioned earlier were acquired for the phantom with the dummy seeds placed. The phantom and its image acquired from the fluoroscopy unit with the dummy seeds in phantom are shown in Figures 2a and 2b respectively.

**Figure 2a:**
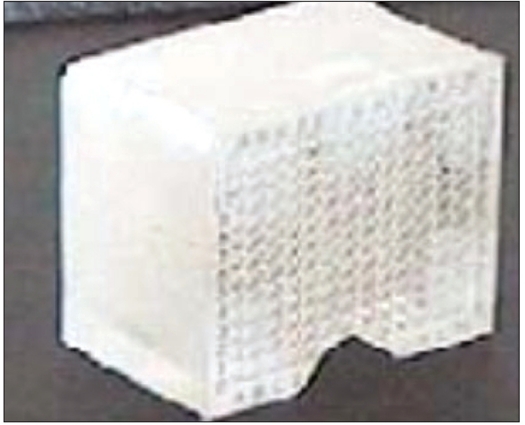
Perspex phantom made to simulate a typical prostate implant

**Figure 2b:**
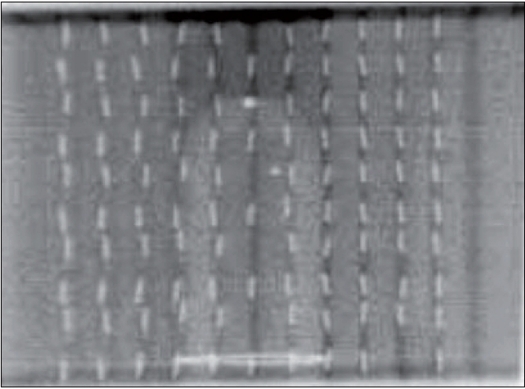
C-arm image of the phantom with 104 seeds

Since the acrylic phantom has a rigid geometry, a gel phantom was also made in order to simulate more closely a seed implant on a patient than the rigid acrylic phantom. This gel phantom was made in an acrylic box of size 15 × 15 × 10 cm^3^. Holes were provided to the geometry of the template used for the implant on the front face of the phantom to enable deposition of the seeds. The box was filled with 4% gel with a colored 10% gel at the middle to simulate a prostate. Provision was also made to place the ultrasound probe below the implant area of the phantom, simulating a trans-rectal ultrasound study. The phantom and the radiographic image of the phantom with seeds are shown in Figures 3a and 3b respectively.

**Figure 3a:**
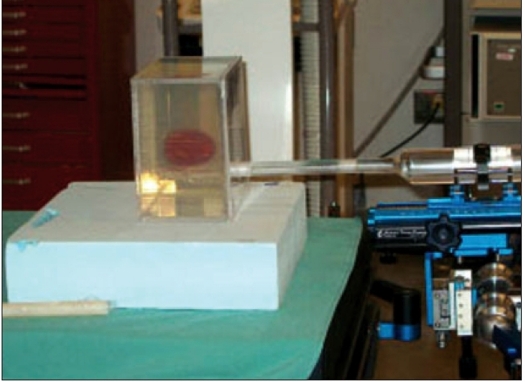
Gel phantom with the dummy probe simulating a prostate implant

**Figure 3b:**
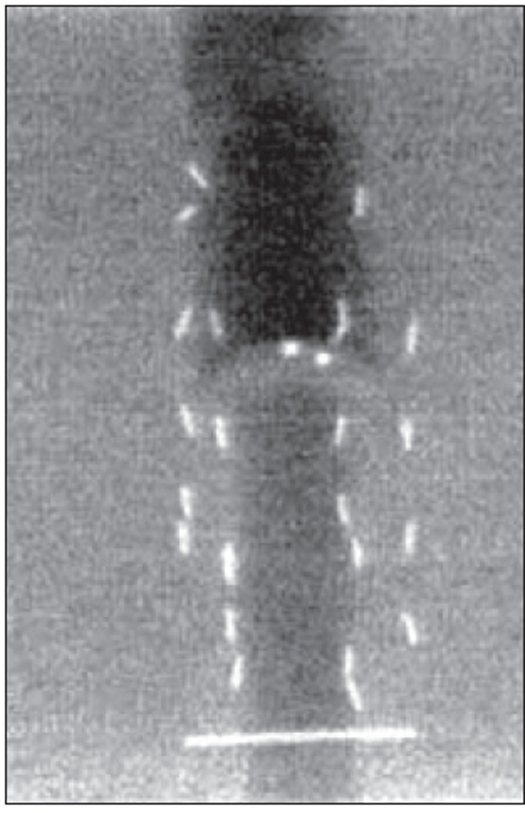
AP C-arm radiographic image of the gel phantom with 24 seeds

### Patient study

To study the suitability of the system developed for intra-operative dosimetry for prostate implant, C-arm fluoroscopic images were acquired at +8°, 0° and −8° during the implant for three patients with the patient in the implant position. The trans-rectal ultrasound probe shadowed the seeds implanted just above the rectum on the radiographs. Hence the ultrasound probe was replaced with an identical dummy probe made of acrylic. This ensured visibility of the seeds and that the reconstructed seed geometry was not disturbed due to the removal of the trans-rectal ultrasound probe. The seed positions were reconstructed with these images and the dose distributions were obtained.

## Results and Discussion

### Phantom study

**Figure 4:**
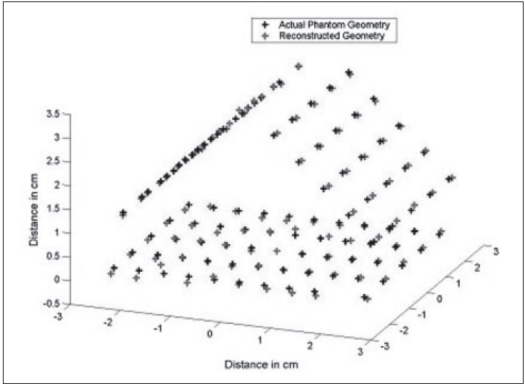
Reconstructed seed position on the acrylic phantom, 104 seeds placed in a triangular geometry

**Figure 5:**
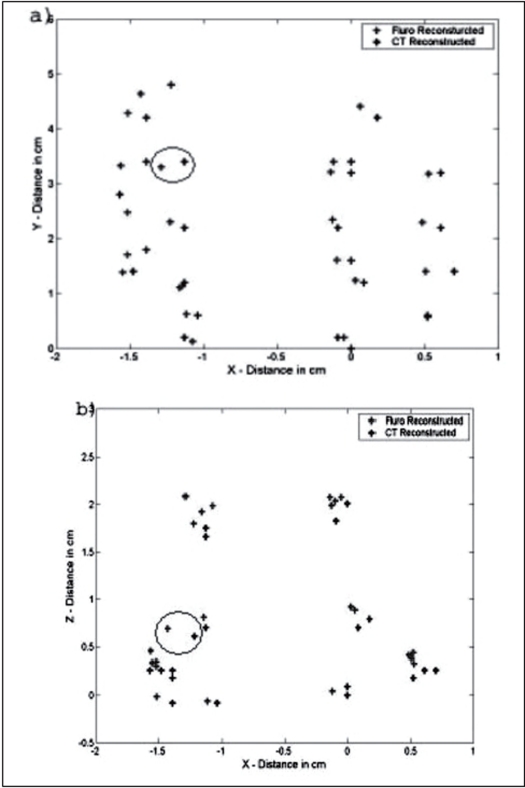
Seed co-ordinates obtained for gel phantom compared with actual seed co-ordinates a) AP view b) Lateral view. The circled seeds show the largest deviations

**Figure 6:**
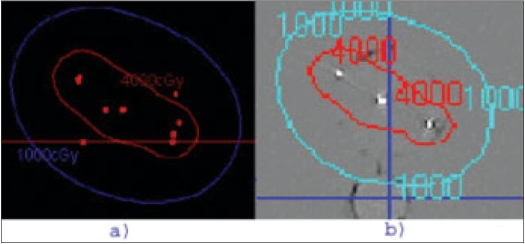
a) Dose distribution obtained for gel phantom implant in the present study. b) dose distribution obtained for the same gel phantom implant with Theraplan Plus planning system

The fluoroscopic images were obtained for the acrylic phantom with 104 seeds placed in a triangular geometry. The seed positions were reconstructed from these images using the software developed in this study and compared with actual seed coordinates. We found that the reconstructed seed coordinates agreed within ±2 mm with the actual seed coordinates in the acrylic phantom. The reconstructed seed positions are compared with the actual positions in Figure 4. This study was repeated by implanting 24 seeds in the gel phantom. The implant in the gel phantom did not result in a perfect geometry and very much resembled an implant on a patient. A CT scan was obtained for this phantom and the reconstructed seed positions in AP obtained from the C-arm images were compared with the coordinates obtained from CT scans of the gel phantom; the maximum deviation observed was ±2-3 mm. This is shown in Figure 5. Dose distribution obtained from the reconstructed geometry was comparable with the dose distribution obtained with the MDS Nordion (now Nucletron) Therplan Plus software for the planned geometry. The dose distribution obtained for the gel phantom implant and the corresponding distribution obtained with Theraplan Plus planning system are shown in Figures 6a and 6b respectively.

### Patient study

A pilot study was performed on patients with the above technique. The fluoroscopic images were acquired for three patients during the implant procedure with the ultrasound probe replaced with the dummy acrylic probe. The seeds were automatically detected and reconstructed using the software developed. A comparison of the reconstructed seed coordinates with the projected anterior coordinates obtained from the film showed a deviation of 5 to 7 mm for a few seeds for the first patient and the investigation on the lateral view showed two seeds were wrongly reconstructed and were totally away from the implant volume. Out of the total 68 seeds, about 95% of the seeds were reconstructed correctly. The AP view and the lateral view are shown in Figures 7a and 7b respectively. For the second patient, an implant was performed with 95 seeds and a comparison of the reconstructed X and Y coordinates with the AP projection showed that the maximum deviation was about 3 to 4 mm for a few seeds and an investigation on the lateral view of the reconstructed coordinates showed that 5 seeds were out of the implant volume. The comparison on the AP projection and the lateral view are shown in Figures 8a and 8b respectively. For the third patient implanted with 82 seeds, the automatic detection resulted in better reconstruction and a comparison of the X and Y coordinates with the AP radiographic projection showed a maximum deviation of only about 2 mm [Figure 9]. The dose distribution was also obtained for the seeds using the software and is shown on the transverse section in Figure 10.

**Figure 7a:**
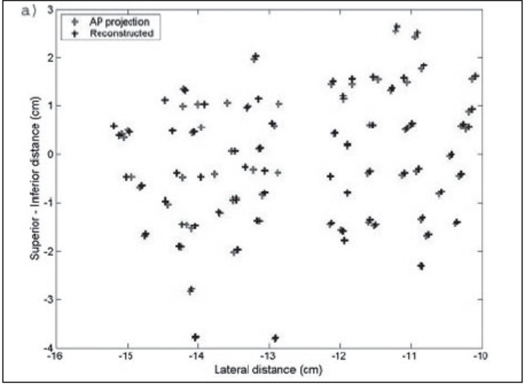
AP view of the reconstructed seed position for patient 1 implanted with 68 seeds, compared with AP coordinates

**Figure 7b:**
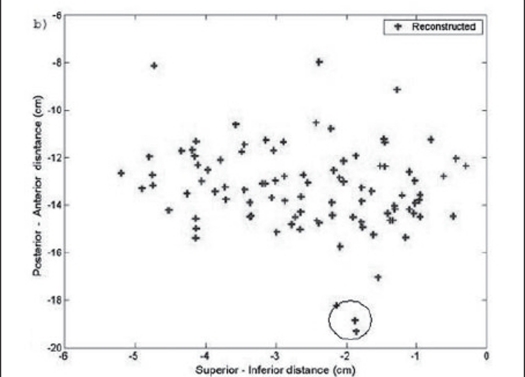
Reconstructed seed position of lateral view. The circle shows seeds reconstructed outside the implant geometry

**Figure 8:**
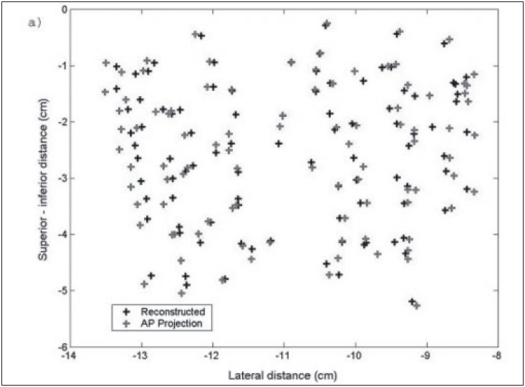
AP Reconstructed seed position for patient 2 implanted with 95 seeds, compared with AP coordinates

**Figure 9:**
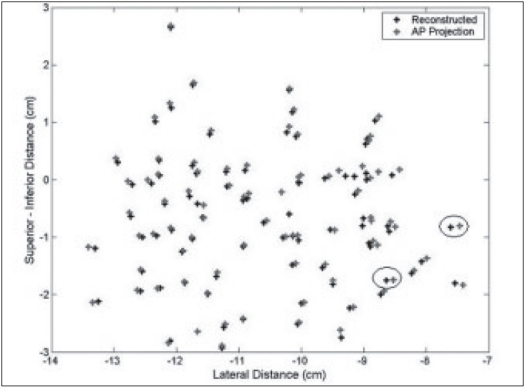
AP Reconstructed seed position for patient 2 implanted with 95 seeds, compared with AP coordinates. The circles show the largest deviation in reconstruction

**Figure 10:**
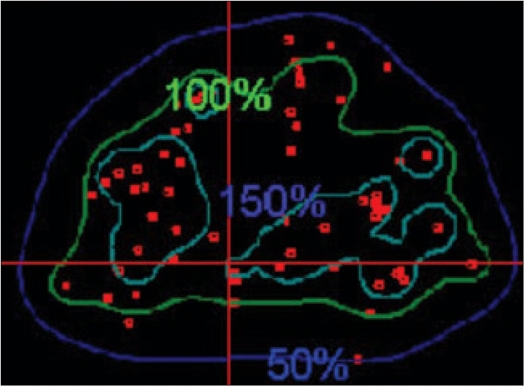
Transverse dose distribution obtained for an implant in the present study

## Conclusion

A dosimetry system with a fluoroscopy unit was developed for intra-operative dosimetry of seed implants performed for prostate cancer. The system was developed with ceiling-mounted C-arm fluoroscopy unit. Phantom studies were performed on an acrylic phantom and on a gel phantom. The phantom studies indicate that it is feasible to use the system to assess the quality of implant. The system was used on three patients and the reconstructed seed positions matched well (within 2 mm) except for few seeds that deviated by 5 to 7 mm. About 95% of the seeds were reconstructed correctly and the results could be further improved with better digital fluoroscopy images.
